# Microscopic polyangiitis secondary to *Mycobacterium abscessus* in a patient with bronchiectasis: a case report

**DOI:** 10.1186/s12890-018-0732-3

**Published:** 2018-11-19

**Authors:** C. Addy, G. Doran, A. L. Jones, G. Wright, S. Caskey, D. G. Downey

**Affiliations:** 1 Centre for Experimental Medicine, Queen’s University Belfast, 97 Lisburn Road, Belfast, BT9 7BL Northern Ireland; 2 Regional Respiratory Centre, Belfast City Hospital, 51 Lisburn Road, Belfast, BT9 7AB Northern Ireland; 3 Department of Respiratory Medicine, Royal Brompton Hospitals, Sydney Street, London, SW3 6NP England; 4 Department of Rheumatology, Musgrave Park Hospital, Stockmans Ln, Belfast, BT9 7JB Northern Ireland

**Keywords:** Non-tuberculous mycobacteria, Non-tuberculous mycobacterial pulmonary disease Mycobacterium abscessus, Vasculitis, Interferon-gamma, Bronchiectasis

## Abstract

**Background:**

Non-Tuberculous Mycobacterial–pulmonary disease (NTM-PD) is increasing in incidence and prevalence. Mycobacterium abscessus (*M.abscessus*) is a rapid growing multi-resistant NTM associated with severe NTM-PD requiring prolonged antibiotic therapy. Complications of therapy are common but reports on direct complications of active NTM-PD are rare. Vasculitis has been described as a rare complication of NTM-PD, most often in individuals with inherited immune defects. This case is the first to describe an ANCA positive vasculitide (Microscopic Polyangiitis) secondary to *M.abscessus* pulmonary disease.

**Case presentation:**

A 70 year old female with bronchiectasis underwent a clinical decline associated with the growth of *M.abscessus* and was diagnosed with NTM-PD. Before treatment could be initiated she developed small joint arthralgia and a glove and stocking axonal loss sensorimotor neuropathy. Positive Perinuclear Anti-Neutrophil Cytoplasmic Antibodies (P-ANCA) and Myeloperoxidase-ANCA (MPO-ANCA) titres led to a diagnosis of microscopic polyangiitis. Further investigation revealed reduced interferon-gamma production but no other significant immune dysfunction.

Dual treatment with immunosuppressive therapy (Corticosteroids/Cyclophosphamide) for vasculitis and antimicrobial therapy for *M.abscessus* NTM-PD was initiated. Clinical stability was difficult to achieve with reductions in immunosuppression triggering vasculitic flares. One flare led to retinal vein occlusion with impending visual loss requiring escalation in immunosuppression to Rituximab infusions. An increase in immunosuppression led to a deterioration in NTM-PD necessitating alterations to antibiotic regimes. Adverse effects including alopecia and Achilles tendonitis have further limited antibiotic choices resulting in a strategy of pulsed intra-venous therapy to stabilise NTM-PD.

**Conclusions:**

This is the first reported case of an ANCA positive vasculitis secondary to *M.abscessus* pulmonary disease. This rare but important complication had a significant impact on the patient adding to the complexity of an already significant disease and treatment burden. The potential role of reduced interferon-gamma production in this case highlights the importance of investigating immune function in those with mycobacterial infection and the intricate relationship between mycobacterial infection and immune dysfunction. Immune dysfunction caused by genetic defects or immunosuppressive therapy is a known risk factor for NTM-PD. Balancing immunosuppressive therapy with prolonged antimicrobial treatment is challenging and likely to become more common as the number of individuals being treated with biologics and immunosuppressive agents increases.

## Background

The incidence and prevalence of Non-Tuberculous Mycobacterial–pulmonary disease (NTM-PD) is increasing [1, 2]. Understanding the spectrum of disease and pathogenicity of differing Non-Tuberculous Mycobacteria (NTM) has advanced in line with developments in microbiological detection highlighting the need to sub-speciate NTM and alter management accordingly [1]. NTM can cause progressive lung disease or reside within the lungs asymptomatically [1]. Treatment regimens are prolonged, toxic and difficult to tolerate. Decisions to commence treatment are based on a number of factors including disease severity, frequency of positive cultures, progression of radiological appearances, underlying lung disease, co-morbidities and pathogenicity of individual species [1].

Guidance on treating NTM-PD was first published in 2007 [3] with no further guidance published until the 2016 Cystic Fibrosis specific consensus document [4] and 2017 British Thoracic Society guidelines [5]. This is due to lack of evidence in this area and to the complexity of the disease. Increasing incidence and prevalence of NTM-PD highlights the need for further research and guidance [1].

Mycobacterium abscessus *(M.abscessus)* is one of the most pathogenic NTMs, particularly in those with underlying lung disease [5]. It is a multi-resistant organism with limited antibiotic options and is challenging to treat [5]. *M. abscessus* is a rapid growing NTM which can be sub-speciated into *M.a.abscessus*, *M.a.massiliense* and *M.a.bolletii* [5]. The initial treatment aim is eradication, which commonly fails and therefore long-term chronic suppressive therapy becomes necessary. Development of macrolide resistance is associated with reduced rates of culture conversion and increased chronic infection [5]. Evidence on optimal antibiotic regimes and longer-term outcomes of chronic suppressive treatment are limited [3, 5]. Those with the subspecies *M.a.abscessus* have much lower rates of culture conversion [5]. The impact of *M.abscessus* infection on patients should not be underestimated because of prolonged toxic treatment regimens that it requires, the need for stringent infection control precautions and it’s relative contra-indication to lung transplantation [4, 5].

## Case presentation

**Fig. 1:**
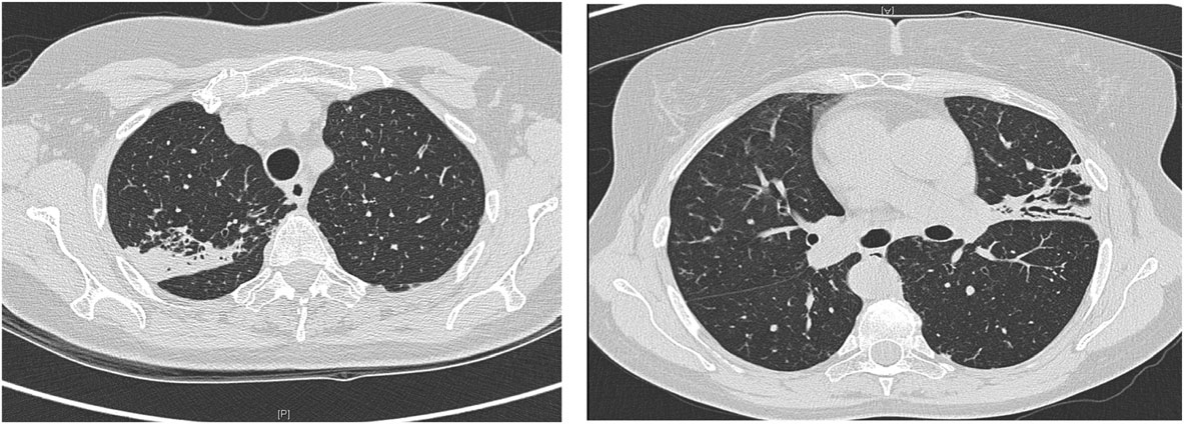
CT Thorax 2007: CT Thorax from 2007 demonstrating bi-apical scarring, right middle lobe atelectasis, right upper lobe cylindrical bronchiectasis and reticulo-nodular densities in both lower lobes

A 70-year-old female had been symptomatic with a persistent non-productive cough and recurrent chest infections for 10 years. At presentation to clinic a CT thorax showed right middle lobe bronchiectasis. She was a life-long non-smoker with no childhood history of respiratory disease and normal baseline investigations for immune deficiency. Clinical stability was achieved with long term Azithromycin and regular airway clearance. *Staphyloccocus aureus (S.aureus)* was cultured intermittently from sputum samples with repeatedly negative mycobacterial cultures. A repeat CT 6 years after her initial scan demonstrated progression of disease with bi-apical scarring, right middle lobe atelectasis, right upper lobe cylindrical bronchiectasis and reticulo-nodular densities in both lower lobes. (Fig. 1) Nebulised Tobramycin was trialled to suppress *S.aureus* and stabilise radiological appearances but stopped after 15 months due to worsening cough.

*M. abscessus* was first isolated from her sputum in November 2013; sub-speciated into *M.a. abscessus*. Commencement of therapy was under consideration when, 3 months from first isolation of *M.abscessus*, paraesthesia developed in her hands and feet with associated small joint arthropathy. A subtle purpuric rash was evident on the lower limbs. Nerve conduction studies confirmed an axonal loss sensorimotor neuropathy. Erythrocyte Sedimentation Ratio was elevated at 49 mm/hr, Perinuclear Anti-Neutrophil Cytoplasmic Antibodies (P-ANCA) titre was highly positive at 80 AI and Myeloperoxidase- ANCA (MPO-ANCA) was > 8 AI with Proteinase-3–ANCA (PR3-ANCA) < 0.2 AI. Renal function and urine microscopy were normal. A diagnosis of microscopic polyangiitis was made. Treatment with Cyclophosphamide and high dose Prednisolone was commenced with full resolution of her paraesthesia.

Within 4 months of first isolation, all sputum samples (*n* = 4) were culture positive and 66% of those samples were smear positive for *M. abscessus*. C-Reactive Protein was elevated at 86.2 mg/l. A repeat CT thorax showed extensive bronchiectasis with underlying collapse in the right upper and lower lobes. There was extensive tree-in-bud change within the right lower lobe, scattered pulmonary nodules and small areas of ground glass shadowing in both lungs. Deteriorating radiological appearances and commencement of active immunosuppression prompted urgent initiation of *M. abscessus* treatment.

### Treatment

Induction phase treatment comprised intravenous (IV) Cefoxitin and Amikacin with oral Clarithromycin and Minocycline and was tolerated for 2 weeks. Long-term nebulised Amikacin was commenced, along with oral Moxifloxacin, Minocycline and Clarithromycin.

A month later she attended the Emergency Department (ED) with rupture of her right Achilles tendon secondary to Moxifloxacin. This was stopped immediately and replaced with Linezolid. Despite dose reduction this was discontinued due to intolerable nausea, diarrhoea and angular stomatitis. She remained on oral Minocycline, Clarithromycin and nebulised Amikacin. Cyclophosphamide was changed to Azathioprine as long-term immunosuppression, but this was poorly tolerated due to nausea. Significant alopecia then developed; attributed to Minocycline, which was also discontinued. Nebulised Meropenem was therefore added to maintain triple antibiotic therapy.

On review 17 months after initiation of treatment her pulmonary disease appeared more stable. A regime of nebulised Meropenem, nebulised Amikacin and oral Clarithromycin was tolerated. Smear and culture negativity was maintained for a further 12 months on this regime. P-ANCA and MPO-ANCA titres remained within normal limits during this period.

**Fig. 2:**
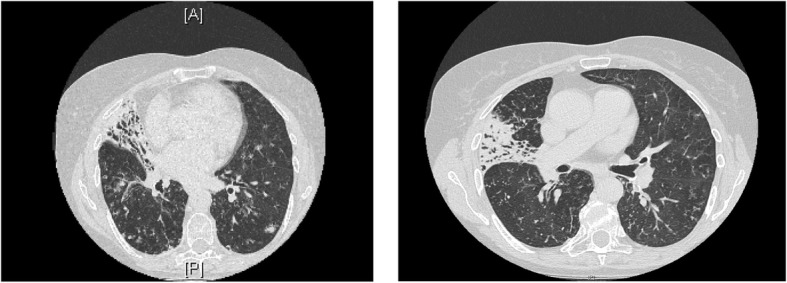
CT Thorax 2016: High resolution CT Chest from 2016 demonstrating extensive airspace opacification, ill-defined nodularity and tree in bud change

Twenty months after starting treatment, cultures again became positive for *M.abscessus*. A month after culture positivity returned, she presented to the ED with visual loss secondary to a right branch retinal vein occlusion. MPO-ANCA had again become highly positive at > 8 AI and P-ANCA titre was 160 AI. Due to active vasculitis with impending visual loss from associated hypercoagulability and thromboembolism higher dose immunosuppression was required. She commenced Rituximab and Methylprednisolone infusions weekly for 4 weeks, followed by 6 monthly Rituximab. Clinical response was achieved with CD19 count reducing from 46% to 0%. Within the following 12 months sputum samples became smear positive again. High resolution CT chest demonstrated extensive airspace opacification, ill-defined nodularity and tree in bud change. (Fig. 2)

### Outcome and follow-up

Further assessment of immunological function was carried out, including vaccination response, respiratory oxidative burst, Mannan Binding Lectin, IgG subsets, complement levels, alternative and innate signalling pathways. Specific testing of interferon pathways demonstrated a very low interferon-gamma (IFN-γ) level, with low production of Interleukin-17 (IL-17) but no autoantibodies to IFN-γ.

No other organisms, including *S.aureus* have been detected by standard sputum culture since 2012. Four years from first isolation she remained *M.abscessus* smear and culture positive. Clarithromycin resistance was now detectable necessitating its cessation. A further 3 weeks of IV Amikacin, Cefoxitin and Tigecycline was commenced. Long-term treatment continued with nebulised Meropenem and Amikacin.

A third oral agent would optimise long-term therapy but intolerances, resistance and risk of visual loss prevent the use of almost all suitable oral agents. Clofazimine was not commenced due to risk of further visual loss. A trial of oral Co-trimoxazole [5] resulted in intolerable nausea and had to be stopped. As no third oral agent could be used, pulsed IV antibiotics have been introduced. The optimal duration between IV courses is unknown and is currently based on recurrence of smear positivity or deteriorating symptoms. The first repeat IV antibiotic course maintained smear negativity for 6 months. Recurrence of smear positivity and associated clinical deterioration have required further IV courses (Table 1).

**Table 1:** Timeline of Case

Time from Presentation	Relevant Past Medical History and Interventions		
Presentation	First review in Respiratory Clinic- recurrent “chest infections” since 1992 Life-long Non-Smoker No childhood History of Respiratory Illness No significant Past Medical History or Family History of note		
	Summary of progress	Diagnostic Tests	Interventions
Presentation	Initial Diagnosis Bronchiectasis Clinically stable	CT thorax Normal Baseline Investigations for immunodeficiency Sputum Culture and Sensitivity testing Sputum for Acid Fast Bacill (AFB) and NTM culture	Airway Clearance regime initiated Maintenance Azithromycin therapy started
Six years from presentation	Increasing frequency of Exacerbations	HRCT thorax: progression of disease with right middle lobe atelectasis, right upper lobe cylindrical bronchiectasis and reticulo-nodular densities in both lower lobes Serial growth of *S. aureus* in sputum samples	Trial of Nebulised Tobramycin- stopped after 15 months due to cough
Eleven years from presentation	First Isolation of *M.abscessus* from Sputum	Further Sputum Samples for AFB/NTM Culture	Eradication therapy under consideration
Three months from first *M.abscessus* culture	Development of paraesthesia in hands, purpuric rash on limbs and small joint arthropathy	ESR 49 P-ANCA highly positive 80AI MPO-ANCA was > 8 AI; PR3-ANCA < 0.2 AI Nerve conduction studies - axonal loss sensorimotor neuropathy Renal Function and Urine microscopy Normal	Diagnosis of microscopic polyangiitis Commenced on immunosuppression-high dose Prednisolone and Cyclophosphamide
Four months from first *M.abscessus* culture	All sputum samples culture positive for *M.abscessus,* 66% samples smear positive	CRP raised at 86.2 mg/l CT thorax: extensive bronchiectasis with underlying collapse in the right upper and lower lobes.	Induction Phase Treatment for *M.abscessus* initiated (IV Cefoxitin/Amikacin/ Oral Clarithromycin/ Minocycline) Maintenance therapy initiated (Nebulised Amikacin,Oral Moxifloxacin/Minocycline/Clarithromycin)
Six months from first *M.abscessus* culture	Attended ED with Achilles tendon rupture	Non-surgical management with immobilization	Moxifloxacin stopped Replaced with Linezolid
Seven months from first *M.abscessus* culture	Developed significant Alopecia		Minocycline Discontinued Nebulised Meropenem added
Two years from first *M.abscessus* culture (Seventeen months into treatment)	Sputum became culture positive for *M.abscessus* Presented to ED with right sided visual loss – 1 month later (18 months into treatment)	Right branch retinal vein occlusion secondary to vasculitis confirmed on fluorescein angiography P-ANCA titre = 160; MPO > 8 AI (highly positive)	Higher level of Immunosuppression initiated (IV Methylprednisolone and 6 monthly Rituximab infusions)
Three years from first *M.abscessus* culture	Clinically stable Smear and culture positive for *M.abscessus*	Further assessment of immunological function: • Very low interferon-gamma (IFN-γ) level, with low production of Interleukin-17(IL-17) but no autoantibodies to IFN-γ. • Normal Vaccination response • Normal Respiratory oxidative burst, Mannan Binding Lectin, Alternative and innate signalling pathways Evidence of efficacy of Rituximab • CD19 count reduced from 46 to 0% • P-ANCA and MPO titres suppressed	Remained on maintenance antibiotic regime Ongoing immunosuppression with Rituximab
Four years from first *M.abscessus* culture	Persistent *M. abscessus* smear and culture positivity	Clarithromycin resistance detectable on sputum culture	Cessation of Clarithromycin Further 3 weeks IV Amikacin/Cefoxitin/Tigecycline. Maintenance nebulised Meropenem and Amikacin
Current (Five years from first *M.abscessus* culture)	Intermittent *M.abscessus* smear positivity Persistent culture positivity	Sensitivity of *M.abscessus* being monitored No growth of other organisms including *S.aureus* in sputum since 2012	Pulsed IV antibiotics at intervals determined by clinical symptoms and smear positivity

## Discussion

This case highlights a rare systemic complication of NTM-PD and is the first reported case of ANCA-positive vasculitis as a consequence of *M. abscessus* infection. It demonstrates the complexity of managing NTM-PD including therapeutic challenges and the relationship between immune function and NTM infection.

NTM-PD is increasing in incidence and prevalence. Determining the clinical relevance of NTM isolates can be difficult [2]. *M. abscessus* is the 3rd most commonly isolated NTM in the USA [5]. It predominantly affects white, female non-smokers over the age of 60 [5]. It can occur in the absence of underlying lung disease where the course can be slow and indolent. In the setting of pre-existing lung disease such as CF or bronchiectasis, it can be fulminant and rapidly progressive [5]. The radiographic picture is of inflammatory nodules, tree-in-bud opacities and cavitation particularly in areas of underlying bronchiectasis [4].

There is wide variation in how patients with *M. abscessus* NTM-PD are treated [4]. An intensive induction phase consisting of three IV antibiotics administered for several weeks in combination with an oral agent is common [4, 5]. This is usually followed by at least two oral antimicrobials in addition to a macrolide antibiotic and inhaled antibiotic(s) [4]. Side effects of antimicrobial therapy are significant and can limit treatment options over a prolonged treatment period. In one study 65% patients stopped at least one antibiotic due to adverse events or toxicity [6]. In this case the additive toxicity of both anti-microbial and immunosuppressive agents further increased the side effect profile (Table 2).

**Table 2:** Adaption from RA Floto, KN Olivier, L. Saiman et al.US Cystic Fibrosis Foundation and European Cystic Fibrosis Society consensus recommendations for the management of non-tuberculous mycobacteria in individuals with cystic fibrosis. Thorax; 2016:71:i1-i22; Haworth CS, Banks J, Capstick T et al. British Thoracic Society Guidelines for the Diagnosis and Management of Non-Tuberculous Mycobacterial Pulmonary Disease (NTM-PD). Thorax; 2017;72:2; Lallana EC and Fadul CE. Toxicities of Immunosuppressive Treatment of Autoimmune Neurologic Diseases. Curr Neuropharmacol. 2011 Sep; 9 (3):468–477 [24]

Drug	Common Side Effects
Amikacin	Nephrotoxicity, Ototoxicity: irreversible vestibulocochlear nerve damage.
Azithromycin	Nausea, Vomiting, Diarrhoea, Fulminant Hepatitis, Pseudomembranous Colitis Ototoxicity Prolonged QT
Clarithromycin	Hepatitis Taste Disturbance Prolonged QT
Cefoxitin	Fever, Rash Eosinophilia, Anaemia, Leucopenia, Thrombocytopenia
Clofazimine	Pink Brownish-Black Discoloration of Skin, Ichthyosis and dry skin Enteropathy, Nausea and Vomiting Conjunctival pigmentation, Dimness of vision and dry eyes.
Co-Trimoxazole	Nausea, Vomiting, Diarrhoea Anaemia, Leucopenia, Thrombocytopenia Hyponatraemia, Hyperkalaemia Fever, Rash, Steven Johnson Syndrome
Ethambutol	Optic Neuritis Hyperuricaemia Peripheral Neuropathy
Imipenem	Rash and Urticaria Hepatitis, Nausea, Vomiting Diarrhoea
Linezolid	Anaemia, Leucopenia, Thrombocytopenia Peripheral Neuropathy Optic Neuritis
Moxifloxacin	Nausea, Vomiting, Diarrhoea Insomnia, Agitation, Anxiety Tendonitis Photosensitivity Prolonged QT
Minocycline	Photosensitivity Nausea, Vomiting, Diarrhoea, Dysphagia Vertigo, Headache Skin Hyperpigmentation
Rifampicin	Orange discoloration of bodily fluids Hepatitis, Nausea, Vomiting, Diarrhoea Fever, Chills Thrombocytopenia Renal Failure Increased hepatic metabolism of numerous drugs
Streptomycin	Nephrotoxicity Ototoxicity
Tigecycline	Nausea, Vomiting, Diarrhoea, Pancreatitis, Bilirubinaemia Increased risk of infections: sepsis/septic shock Hypoproteinaemia
Corticosteroids	Cataract Increased risk of infection Type 2 Diabetes Mellitus Osteoporosis Gastrointestinal bleeding Altered Mood – rarely psychosis Proximal myopathy Hypertension
Cyclophosphamide	Leucopenia Haemorrhagic Cystitis Infertility Long term increase risk of cancer e.g. Bladder Malignancy, Myeloproliferative disorders Nausea, Hair Loss, Skin Irritation
Azathioprine	Hepatotoxicity, Pancreatitis Leucopenia Fatigue, Hair loss Diarrhoea Increased risk of malignancy
Rituximab	Rash GI upset Serious Infection, CNS toxicity

The association between vasculitis and chronic suppurative lung conditions [7, 8] is thought to result from excessive humoral immune responses secondary to circulating immune complexes [8]. Pulmonary infection may trigger vasculitis through induction of ANCA antigen expression on the surface of neutrophils [9]. The temporal relationship between *M.abscessus* growth, ANCA positivity and vasculitic symptoms reduces the chance of the two being unrelated, suggesting *M.abscessus* was a causative trigger for the vasculitides. *S.aureus* had been chronically cultured since presentation with no prior evidence of vasculitis reducing the likelihood of *S.aureus* being a causative factor. Previous case reports have described ANCA-positive vasculitis secondary to *M.avium complex* [10]. Leukocytoclastic vasculitis linked to Salmonella has been reported in children with genetic Interferon-gamma deficiency [11]. Leukocytoclastic vasculitis in an individual with interferon-gamma autoantibodies and disseminated *M. abscessus* has also been reported [12]. Increased susceptibility to mycobacterial disease has been reported in genetic interferon defects but a causal link with associated vasculitides has not been established [13]. It is possible the reduction in IFN-γ seen in this case may have contributed both to development of vasculitis and poor treatment response.

The use of IFN-γ had been considered in this case. IFN-γ production is integral to mycobacterial defence through activation of both innate and adaptive immune systems via the interleukin12-IFN-γ pathway [13, 14]. IFN-γ facilitates mycobacterial killing by enhancing phagocytosis and expression of oxygen free radicals [14]. Administration is associated with a flu like syndrome [15–17]. Intramuscular IFN-γ as adjuvant therapy for *M. avium complex* resulted in clinical benefit over antibiotics alone [5, 15] but of insufficient magnitude to warrant recommending routine use [5]. IFN-γ therapy in management of *M. abscessus* disease is limited to case reports of use in disseminated disease [16, 18].

Rituximab has been successfully used in disseminated *M. abscessus* disease refractory to antibacterial chemotherapy in those with IFN-γ autoantibodies [12, 19]. It can restore IFN-γ signalling in these individuals [12, 19]. Whether this is true in those without autoantibodies, as in this case is unknown, as is any potential interaction between Rituximab and IFN-γ therapy. In this case monitoring Rituximab response first was considered of lowest risk but the addition of IFN-γ could be re-visited.

Treatment regimens for systemic vasculitis comprise high dose induction immunosuppression followed by oral maintenance therapy for 12–18 months [9]. The challenge in this case was balancing adequate immunosuppression with sufficient antimicrobial therapy to maintain clinical stability alongside smear and culture negativity. Reductions in immunosuppression triggered vasculitic flares with sight threatening consequences whilst increases in immunosuppression led to a deterioration in NTM-PD.

This case highlights the complex interplay between immune function and NTM-PD both as a complication and a cause of disease [13, 14, 20]. Defects in IFN-γ pathways, both acquired and genetic are associated with NTM-PD [14, 20]; as are defects in macrophage and dendritic cell function and cytokine signalling [5, 13]. Whole genome sequencing has demonstrated higher rates of genetic variants in immune, Cystic Fibrosis Transmembrane Conductance Regulator, cilial function and connective tissue categories in people with NTM-PD. [21] Screening for immune defects in those with NTM-PD who fail to respond to “standard” treatment is increasingly recommended [5] and may allow better prognostication and management in the future. Equally, those with known immune dysfunction should be screened for NTM.

Striking the balance between managing infection and immunosuppression is an increasing issue in clinical medicine with rising numbers of patients receiving biologics, immunosuppressive agents and increasing lifespan post haematological and solid organ transplants [5, 22, 23]. Oral corticosteroid use is up to eight times higher amongst cases of NTM infection [22]. Rates of NTM-PD are five to ten fold higher in patients on anti-TNF alpha therapies [23]. Whilst other biological agents, carry a theoretical increased risk of NTM there is little data on infection rates [15]. Acquisition of NTM infection is likely due to disruption or depletion of cell mediated immunity which is a critical component of host defence against mycobacterium [14]. Adequate screening of high-risk populations for NTM is necessary to further understanding of disease rates and facilitate appropriate intervention [1, 5, 22] (Table 3).

**Table 3:** Key Learning points and Patient Perspective

Patient Perspective “When I was diagnosed with bronchiectasis in my late forties, it did not affect me day to day and I continued to work, raise my family and have an active social life. However, since being diagnosed with *Mycobacterium Abscessus* and Vasculitis, I have struggled with tiredness and progressive shortness of breath especially when exerting myself. I am no longer as active as I wish to be, in particular I struggle to keep up with my toddler grand-daughter and make it around the golf course. However, I make the effort to continue to take light exercise every day by meeting friends for coffee, going shopping and short walks when the weather permits.” Learning Points • NTM Pulmonary disease is increasing in prevalence and should be screened for in in at risk groups. NTM-PD should also be considered if appropriate radiological appearances and /or symptoms develop in these groups. • Treatment regimens are toxic and difficult to tolerate and therefore timing of treatment and goals of treatment are important as well as clear communication with patients in this regard • With increases in therapies which impair immunity the rates of NTM-PD in these groups may continue to rise • Vasculitis triggered by NTM is a rare but significant complication of NTM and this is the first reported case of this being due to M.Abcessus. • Balancing effective immunosuppression with active treatment for infection is challenging and requires specialist expertise and collaborative working between specialist services.	

## Conclusion

Effective treatment of NTM-PD requires effective communication and team working between specialities treating this complex group of patients. Balancing immunosuppressive with anti-microbial regimes and their relative toxicities is challenging and requires frequent assessment, monitoring and treatment adjustment. Further research will refine management approaches and improve our understanding of the role of innate, adaptive and auto-immune dysfunction and the incidence of related complications including vasculitis.

## Abbreviations

AFB: Acid Fast Bacilli
ANCA: Anti-Neutrophil Cytoplasmic Antibodies
CT: Computed Tomography
ED: Emergency Department
IFN-γ: Interferon – gamma
IL-17: Interleukin-17
IV: Intra-venous
*M.a.abscessus*: Mycobacterium abscessus abscessus
*M.a.bolletii*: Mycobacterium abscessus bolletii
*M.a.massiliense*: Mycobacterium abscessus massiliense
*M.abscessus*: Mycobacterium abscessus
MPO-ANCA: Myeloperoxidase Anti-Neutrophil Cytoplasmic Antibodies
NTM: Non-Tuberculous Mycobacteria
NTM-PD: Non-Tuberculous Mycobacterial Pulmonary disease
P-ANCA: Perinuclear Anti-Neutrophil Cytoplasmic Antibodies
PR3-ANCA: Proteinase − 3- Anti-Neutrophil Cytoplasmic Antibodies
*S.Aureus*: Staphyloccocus aureus
TNF-alpha: Tumour Necrosis Factor - alpha
